# Surgical treatment of tricuspid regurgitation after mitral valve surgery: a retrospective study in China

**DOI:** 10.1186/1749-8090-7-30

**Published:** 2012-04-10

**Authors:** Zong-Xiao Li, Zhi-Peng Guo, Xiao-Cheng Liu, Xiang-Rong Kong, Wen-Bin Jing, Tie-Nan Chen, Wan-Li Lu, Guo-Wei He

**Affiliations:** 1 Department of Cardiovascular Surgery, TEDA International Cardiovascular Hospital, Medical College, Nankai University, Tianjin, China; 2 Department of Surgery, Oregon Health & Science University, Portland, OR 97225, USA; 3 Department of Cardiovascular Surgery, TEDA International Cardiovascular Hospital, Medical College, Nankai University, No.61, the Third Avenue, TEDA, Tianjin, China 300457

**Keywords:** Tricuspid regurgitation, Annuloplasty, Tricuspid valve replacement, Mitral valve surgery, Rheumatic heart disease

## Abstract

**Background:**

Functional tricuspid regurgitation (TR) occurs in patients with rheumatic mitral valve disease even after mitral valve surgery. The aim of this study was to analyze surgical results of TR after previous successful mitral valve surgery.

**Methods:**

From September 1996 to September 2008, 45 patients with TR after previous mitral valve replacement underwent second operation for TR. In those, 43 patients (95.6%) had right heart failure symptoms (edema of lower extremities, ascites, hepatic congestion, etc.) and 40 patients (88.9%) had atrial fibrillation. Twenty-six patients (57.8%) were in New York Heart Association (NYHA) functional class III, and 19 (42.2%) in class IV. Previous operations included: 41 for mechanical mitral valve replacement (91.1%), 4 for bioprosthetic mitral valve replacement (8.9%), and 7 for tricuspid annuloplasty (15.6%).

**Results:**

The tricuspid valves were repaired with Kay's (7 cases, 15.6%) or De Vega technique (4 cases, 8.9%). Tricuspid valve replacement was performed in 34 cases (75.6%). One patient (2.2%) died. Postoperative low cardiac output (LCO) occurred in 5 patients and treated successfully. Postoperative echocardiography showed obvious reduction of right atrium and ventricle. The anterioposterior diameter of the right ventricle decreased to 25.5 ± 7.1 mm from 33.7 ± 6.2 mm preoperatively (P < 0. 05).

**Conclusion:**

TR after mitral valve replacement in rheumatic heart disease is a serious clinical problem. If it occurs or progresses late after mitral valve surgery, tricuspid valve annuloplasty or replacement may be performed with satisfactory results. Due to the serious consequence of untreated TR, aggressive treatment of existing TR during mitral valve surgery is recommended.

## Background

Mitral valve replacement (MVR) has been the most common surgical procedure for rheumatic mitral valve disease including stenosis and incompetence. Functional tricuspid regurgitation (TR) is frequently associated with rheumatic mitral valve disease in the patients undergoing MVR. In addition, in patients who had previous mitral valve replacement, TR is also a frequently encountered complication.

Which patient undergoing mitral valve surgery should also have the tricuspid repair is an important clinical question [1]. It was proposed to treat TR independently from the grade of regurgitation when the annular dimension is over 21 mm/m^2 ^or more than or equal to 3.5 cm at echocardiography measurement or when the intra-operative tricuspid annulus (TA) diameter is > 70 mm [1], although others suggested that echocardiographic tricuspid annular dimensions alone, in the absence of significant (less or equals to 1+) TR preoperatively, should not dictate the performance of tricuspid repair [2]. However, for TR after MVR, the opinion for treatment is still in controversy. Most recently, Rogers and associates [3] suggested that if untreated at the time of surgical mitral valve repair, significant residual TR negatively impacts perioperative outcomes, functional class, and survival and that TR does not reliably resolve after successful mitral valve surgery. Further, if present at the time of mitral valve surgery, TR can usually be effectively addressed with ring annuloplasty. Because reoperations for recurrent TR carry high mortality rates, few patients are offered reoperation for redo tricuspid repair or replacement.

We therefore, in the present study, report our experience in surgical treatment of TR post-mitral surgery.

## Methods

### Clinical data

From September 1996 to September 2008, a consecutive series of 45 patients who had second operation for TR after mitral valve replacement in the TEDA International Cardiovascular Hospital and previously incorporated hospitals were retrospectively enrolled in this study. The patients who had concomitant surgery apart from mitral valve replacement at the first operation or during the second operation for the TR were excluded from the present study. There were 12 male (26.7%) and 33 female patients with median age of 49 year-old, ranging 27-69 years. All patients were diagnosed rheumatic heart disease. Forty-three patients (95.6%) had right heart failure symptoms (edema of lower extremity, ascites, hepatic congestion, etc.). Forty patients (88.9%) had persistent atrial fibrillation. Only 5 patients were in sinus rhythm (11.1%). Patients who had severe TR by echocardiography (24 patients, 53.3%) had clinical TR. Twenty-six patients (57.8%) were in New York Heart Association (NYHA) functional class III, and 19 (42.9%) in class IV. The chest X ray showed that all patients had enlarged heart with the cardiothoracic ratio more than 55%. The average time from the pervious operation was 6.8 ± 3.4 years ($\bar{x}\pm \text{SD}$). The previous operations included: mechanical mitral valve replacement (41, 91.1%), bioprosthetic mitral valve replacement (4, 8.9%), and tricuspid annuloplasty (7, 15.6%).

All patients were investigated preoperatively by means of Doppler echocardiography. In our grading system, mild TR = grade 1; moderate TR = grade 2 and 3; severe TR = grade 4. Echocardiography showed moderate TR in 21 cases (46.7%) and severe TR in 24 patients (53.3%).

The systolic pressure of pulmonary artery was 57.3 ± 19.6 mmHg ($\bar{x}\pm \text{SE}$).

Common laboratory findings in those patients were: elevation of transaminase (in 40 patients, 88.9%), hypoalbuminemia and anemia (in 4 patients, 8.9%). Blood urea nitrogen and creatinine increased in 1 case (2.2%).

All patients received digoxin and diuretic therapy to improve the cardiac function preoperatively. The heart rates were controlled by medications in those patients with rapid atrial fibrillation. The patients with anemia (Hb < 8 g/L) or hypoproteinemia were transfused with red blood cells or albumin. The Characteristics of the patients are listed in Table 1.

**Table 1:** Patient Characteristics

Sex	
Male	12

Female	33

**Rheumatic Heart Disease**	

	45

**NYHA Class**	

I-II	0

III	26

IV	19

**Atrial fibrillation**	

	40

**Mitral Valve Replacement (first op.)**	

Mechanical	41

Bioprosthetic	4

**Tricuspid Annuloplasty (first op.)**	

	7

**Tricuspid Regurgitation**	

Moderate	21

Severe	24

**Preoperative Medicine**	

Digoxin	45

Furosemide	11

Hydrochlorothiazide	35

Spironolactone	44

**Tricuspid Operation**	

Annuloplasty	

De Vega	4

Kay	7

Replacement	

Mechanical	22

Bioprosthetic	12

*Af *: atrial fibrillation

### Surgical procedures

All patients underwent the operation with general anesthesia and intubation. The sternotomy was performed with swing saw with the adhesion carefully dissociated and the wires removed. Cardiopulmonary bypass was prepared routinely. The surgical procedures were either tricuspid annuloplasty or valve replacement. The decision to perform valve replacement was mainly due to the preference of the patient and the surgeon (XRK). Unlike in the Western countries, in China most patients prefer "one-surgery-only" procedures to avoid future operations. In this experience, most tricuspid valve replacements were performed due to the request of the patient by one surgeon (XRK) while the annuloplasty was performed by others.

Tricuspid annuloplasty was performed with annuloplasty, either semicircular (classical or modified De Vega repair, 4 cases) or simple lateral annuloplasty (Kay repair, 7 cases). Due to cost of the tricuspid annuloplasty ring and the extensive experience on tricuspid repair in the primary tricuspid valve surgery by the surgeons (XCL, WBJ, and GWH) in this group, no rings were used in this experience.

Thirty-four patients had valve replacement with either mechanical (22) or bioprosthesis (12). The details of the prosthesis used are listed in Table 2.

**Table 2:** The details of the prostheses used for tricuspid valve replacement during the re-operation

	Mechanical prosthesis (ATS)					Bioprosthesis (BalMedic)				
Size (mm)	23	25	27	29	31	23	25	27	29	31

Patient number	0	0	13	9	0	0	0	7	5	0

### Statistical analysis

Data were described as mean ± standard deviation ($\bar{x}\pm \text{SD}$), or median [quartiles] (median [25%, 75%]). Paired *t*-test was used to compare the pre- and post-operative cardiac function. The data were analyzed with ANOVA by using SPSS 13.0. *P *< 0.05 was considered statistically significant.

## Results and discussion

As described above, the tricuspid annuloplasty was performed in 11 patients with Kay's (7 cases, 15.6%) or De Vega technique (4 cases, 8.9%).

In patients received tricuspid valve replacement (34 cases, 75.6%), 2 cases initially had annuloplasty that was demonstrated unsatisfactory by intraoperative transesophageal echocardiography after the repair and tricuspid valve replacement was then immediately performed.

One patient who had tricuspid valve replacement died (2.2%) due to severe left ventricular dysfunction that caused refractory low cardiac output postoperatively. There was no mortality in the tricuspid repair group. In addition, postoperative low cardiac output syndrome occurred in other 5 patients that were treated successfully. Regarding the occurrence of low cardiac output, there were no differences between the tricuspid valve replacement group (4/34, 11.8%) and tricuspid repair group (1/11, 9.1%, p > 0.05). There were no differences between the two groups with regard to the hospital stay, inotropic support, and pre- and post-operative NYHA class.

Before discharge, postoperative echocardiography showed significant reduction of the right atrium and ventricle in all patients. The anterioposterior diastolic diameter of the right ventricle decreased to 23-41 mm (25.5 ± 7.1 mm), comparing with the preoperative data of 31 ~ 48 mm (33.7 ± 6.2 mm, *P *< 0. 05).

### Follow-up

Owing to the fact that some patients were from remote rural areas and that due to the rapidly growing economy in China the habitation of the rural population is frequently and uncontrollably movable, follow-up on this group of patients was extremely difficult. All patients were followed at 6 months with echocardiography at either our outpatient clinic or the local hospital. However, there were only 25 patients successfully followed beyond 1.5 years. The average follow-up period was 6.8 ± 3.4 years, ranging from 1.5-12 years.

**Figure 1:**
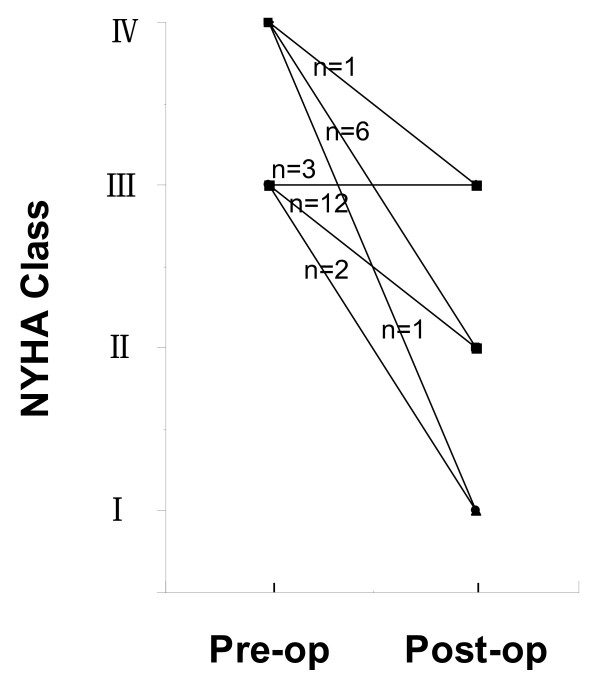
**Comparison between the preoperative cardiac function and the cardiac function at the follow-up**. NYHA Class: New York Heart Association functional class.

At the follow-up, all patients had improvement in cardiac function (Figure 1). The average NYHA functional class was significantly improved from preoperative 3.32 ± 0.10 to 2.04 ± 0.11 (p < 0.0001). As shown in the Figure 1, 8 patients who were in NYHA Class IV preoperatively were in class I, II, or III during the follow-up. Similarly, 14 of 17 patients who were in NYHA class III preoperatively were in class I or II at the follow-up.

Treatment of TR after mitral valve replacement is a rather complicated issue. First, the TR could be residual one that presented at the first operation but not treated during the operation. Second, the TR could be the newly-developed one that was due to continuous pathological progress after the mitral surgery.

Although the short-term outcome of mitral valve replacement without dealing with TR may be satisfactory, unacceptably high rates of residual and/or recurrent TR at the mid- and long-term remain a problem for clinical challenging.

The repair of TR at the first mitral operation is an interesting topic. In the present study, only 7 patients (15.6%) received tricuspid annuloplasty in the previous operation. This is probably due to the reason that in the early period it was a general belief that mild and moderate TR, either of functional or of organic cause, would be improved after surgical correction of left-side valvular lesions [4]. However, it was reported later that the incidence of severe TR post mitral valve operation is about 10%-16% [5,6], despite sometimes no obvious TR was observed during the previous operation. In fact, Matsunaga and associates investigated 70 patients who underwent mitral valve repair and found that the incidence of TR increased from 25% at 1 year to 53% between 1 and 3 years, and 74% at 3 years [7]. Similarly, Dreyfus and colleagues found that in about 50% of the cases of mitral valve repair the tricuspid annulus was abnormally dilated, even in the absence of TR. In the late follow-up, the patients who did not have a tricuspid annuloplasty had progressive tricuspid dysfunction. Therefore, they recommended "prophylactic" tricuspid annuloplasty [8].

The causes of functional TR after mitral valve surgery may include: (1) continuing expansion of right ventricle and tricuspid annulus; (2) persistent pulmonary artery hypertension that may cause right ventricular dilatation and dysfunction [9]; (3) residual stenosis and insufficiency of mitral valve lead to functional TR; (4) myocardial fibrosis in patient with rheumatic heart disease that can aggravate the right ventricular dysfunction, which is followed by TR; (5) severe cardiac arrhythmia that worsens the right ventricular function; (6) right ventricular dysfunction following intraoperative or postoperative ventricular ischemia; and (7) the presence of a trans-tricuspid pacemaker lead that is another known factor for late TR development secondary to adhesions and fibrous retraction [1].

Based on the above reasons, GoTo and colleagues believed that tricuspid insufficiency with advanced mitral valve disease, even of a slight degree, should be surgically treated and that annuloplasty has more obvious hemodynamic benefits than valve replacement [10]. It is now more or less the commonly accepted concept that aggressive treatment of TR during the first valve surgery would benefit the cardiac function, particularly the right heart function, for the long term [10,11].

In this study, 43 patients (95.6%) had right heart failure symptoms and the majority of patients were in atrial fibrillation. Similarly, most patients were in New York Heart Association NYHA functional class III or IV. These data indicated the severity of the condition of the patient with TR post-mitral surgery. Furthermore, even long-period medical therapy could not release the symptoms and improve the quality of life in those patients and therefore an aggressively surgical treatment was strongly indicated.

From the present study, we would like to emphasize that it is necessary to treat the patient with proper medications preoperatively (see Table 1 for details)in order to improve cardiac function particularly the right ventricle function and the general condition of the patient. Such preparations are necessary in order to reduce the incidence of post-operative low output syndrome or other complications.

The surgical procedure of correction of TR is usually the surgeon's preference. Several techniques are available to correct functional tricuspid regurgitation [12]. The recommended methods include: the tricuspid annuloplasty by using simple stitches, such as semicircular (classical or modified De Vega repair) or simple lateral annuloplasty (Kay); novel techniques such as edge-to-edge or clover technique and suture bicuspidization technique; use of flexible and rigid prosthetic rings or 3D rings; flexible prosthetic bands; and use of artificial chordae with polytetrafluoroethylene sutures for anterior and septal tricuspid leaflet pathology [12,13].

Mangoni et al. [14] reported a high mortality after isolated tricuspid valve replacement. We found that our study had a different population compared with theirs. They reported "undergoing isolated tricuspid valve replacement from 1984 to 1996. The cause of valve dysfunction was rheumatic heart disease in 12 patients, healed endocarditis in two patients, and sarcoidosis in one patient." They dealed with a very rare group of patients with tricuspid valve diseases who did not have mitral valve diseases. In contrast, our patients were those who underwent mitral valve replacement at the first instance and then had the second operation for severe tricuspid regurgitation. Similarly, Kim et al. [15] reported 61 patients with isolated severe tricuspid regurgitation undergoing corrective surgery and they had 10% hospital mortality and 3 patients died during follow-up. Again, the patient's population of Kim and colleagues was different from ours but similar to the patient population of Mangoni and associates. Clearly the different results obtained by us and by Mangoni or Kim and their colleagues were mainly due to completely different patient populations.

The landmark studies on the tricuspid valve surgery [16,17] reviewed two series that had higher mortality, indicating the seriousness of the patient conditions and the superiority of the annuloplasty ring over simple sutures [16]. In the present study, we had low mortality compared to their series. This is partially because of the different patient populations. McCarthy and colleagues described a severe TR in their patients for the first operation whereas our patients usually had less TR during the first operation but the TR was more severely developed after the mitral surgery. Nevertheless, the advancement of cardiopulmonary and operative technique must also played a role since our patients were operated more recently than the patients in the series by Bernal and colleagues [17] who were operated Between 1976 and 2002.

Most tricuspid valve replacements were performed due to the request of the patient and the surgeon's preference that also played a role since most of tricuspid valve replacement was performed by one surgeon (XRK) while others preferred tricuspid annuloplasty. Further, in our most recent practice, we (ZXL, XCL, WBJ, and GWH) prefer annuloplasty in the tricuspid position even in the second operation. This principle is actually more acceptable most recently [18]. As reported by Singh and colleagues, tricuspid valve repair is associated with better perioperative, midterm, and event-free survival than replacement in patients with organic tricuspid disease. We agree that repair should be performed whenever possible in patients with organic tricuspid disease [18].

Similarly, the choice of the prosthesis was also the preference of the patient. In China, again, due to the economic status, many patients request to have mechanical prostheses for the durability and this is why more than half of the patients received mechanical prostheses in this experience.

From the present study, we believe that after mitral valve surgery, for those patients with moderate TR, moderate pulmonary hypertension, and without severe dilatation of tricuspid valve annulus, tricuspid annuloplasty is indicated. However, if the patient has severe TR and symptoms of heart failure before operation, or has had annuloplasty in the previous surgery, valve replacement may be considered. Postoperative echocardiography showed excellent results of these procedures with significant reduction of the right atrium and ventricle and improvement of cardiac function.

### Limitation of Study

This study was composed of the experience from a group of surgeons who have their own preference for tricuspid valve replacement or repair, and for the method of repair. Therefore, the uniform opinion as to the indication for tricuspid replacement or repair was not formed although all surgeons in this group agree that for the significant tricuspid regurgitation at mitral valve surgery and for the recurrent tricuspid regurgitation developed after the mitral surgery, the active surgical treatment is necessary. Further, due to the geographic limitations, the follow-up is limited. To reach the definite conclusion for the procedure to treat the tricuspid regurgitation, longer follow-up would be essential in the future.

## Conclusions

In summary, TR after mitral valve replacement in rheumatic heart disease is a serious clinical problem. If it occurs or progresses late after mitral valve surgery, active surgical treatment should be indicated with either tricuspid valve annuloplasty or replacement that may lead to satisfactory results. Due to the serious consequence of untreated TR, aggressive treatment of existing TR during mitral valve surgery is recommended.

## Competing interests

The authors declare that they have no competing interests.

## Authors' contributions

ZXL assisted most of the operations, collected and prepared the data and participated in writing. ZPG participated in collection and preparation of the data as well as in writing. XCL, XRK, and WBJ performed some of the operations and contributed to preparation of the manuscript. TNC and WLL participated in operations as well as preparation of the data. GWH performed some of the operations, designed the retrospective study, and participated in data analysis, manuscript writing, and submission. All authors read and approved the final manuscript.
